# VaxBot-HPV: A GPT-based Chatbot for Answering HPV Vaccine-related Questions

**DOI:** 10.21203/rs.3.rs-4876692/v1

**Published:** 2024-09-11

**Authors:** Cui Tao, Yiming Li, Jianfu Li, Manqi Li, Evan Yu, Muhammad Amith, Lu Tang, Lara Savas, Licong Cui

**Affiliations:** Mayo Clinic; The University of Texas Health Science Center at Houston; Mayo Clinic; The University of Texas Health Science Center at Houston; The University of Texas Health Science Center at Houston; The University of Texas Medical Branch at Galveston; Texas A&M University; The University of Texas Health Science Center at Houston; The University of Texas Health Science Center at Houston

**Keywords:** Vaccine, HPV vaccine, Cervical Cancer, GPT, Large Language model, QA system, Chatbot, Medical education

## Abstract

**Background:**

HPV vaccine is an effective measure to prevent and control the diseases caused by Human Papillomavirus (HPV). This study addresses the development of VaxBot-HPV, a chatbot aimed at improving health literacy and promoting vaccination uptake by providing information and answering questions about the HPV vaccine;

**Methods:**

We constructed the knowledge base (KB) for VaxBot-HPV, which consists of 451 documents from biomedical literature and web sources on the HPV vaccine. We extracted 202 question-answer pairs from the KB and 39 questions generated by GPT-4 for training and testing purposes. To comprehensively understand the capabilities and potential of GPT-based chatbots, three models were involved in this study : GPT-3.5, VaxBot-HPV, and GPT-4. The evaluation criteria included answer relevancy and faithfulness;

**Results:**

VaxBot-HPV demonstrated superior performance in answer relevancy and faithfulness compared to baselines (Answer relevancy: 0.85; Faithfulness: 0.97) for the test questions in KB, (Answer relevancy: 0.85; Faithfulness: 0.96) for GPT generated questions;

**Conclusions:**

This study underscores the importance of leveraging advanced language models and fine-tuning techniques in the development of chatbots for healthcare applications, with implications for improving medical education and public health communication.

## 1. Introduction

Human Papillomavirus (HPV) is a group of viruses that infect the skin and mucous membranes, with over 100 types identified [1]. HPV is primarily transmitted through sexual contact and can infect the genital area, leading to genital warts and various cancers, including cervical, anal, penile, vaginal, vulvar, and oropharyngeal cancers [2], [3], [4]. Among these, cervical cancer stands out as the most common HPV-related cancer and a leading cause of cancer-related deaths in women worldwide, contributing to an estimated 266,000 cervical cancer deaths annually due to HPV infection [5], [6], [7], [8], [9]. This burden is especially pronounced in low- and middle-income countries where access to screening and treatment is limited [5].

Similar to other infectious diseases, the development of HPV vaccines has also been a significant advancement in preventive healthcare [10], [11], [12]. HPV vaccines primarily target HPV types 16 and 18, which are responsible for approximately 70% of cervical cancers and a significant proportion of other HPV-related cancers [13]. By preventing HPV infection, these vaccines can effectively reduce the incidence of HPV-related diseases, including cervical cancer [13]. Clinical trials have demonstrated the high efficacy of HPV vaccines in preventing HPV infection and related diseases [14]. Furthermore, population-based studies have shown a substantial decline in HPV infections and HPV-related outcomes in countries with high HPV vaccination coverage, highlighting the real-world effectiveness of these vaccines [15]. Overall, HPV vaccines are a crucial tool in the prevention of HPV-related diseases, particularly cervical cancer. Widespread vaccination has the potential to significantly reduce the burden of HPV-related cancers and improve the overall health outcomes of populations globally [16].

Despite the proven benefits of HPV vaccination, there are various concerns and forms of hesitancy surrounding its use [17]. Some individuals and communities are hesitant due to insufficient and inadequate information about HPV vaccination or misinformation about the vaccine’s safety and efficacy, often fueled by misinformation spread through social media and other channels [18], [19], [20]. Concerns about the long-term effects of the vaccine and its perceived necessity for individuals who may not consider themselves to be at high risk for HPV-related diseases also contribute to hesitancy [20]. Additionally, cultural or religious beliefs, distrust of pharmaceutical companies, and concerns about the vaccination’s affordability and accessibility in low-resource settings can all play a role in vaccine hesitancy [21]. Addressing these concerns through accurate information, targeted education campaigns, and improved access to vaccination services is crucial in increasing HPV vaccination rates and reducing the burden of HPV-related diseases.

Traditionally, question answering (QA) systems have been developed using rule-based approaches, information retrieval techniques, deep learning-based approaches, or hybrid methods [22], [23]. Rule-based QA systems rely on predefined rules and patterns to extract relevant information from a knowledge base or document collection in response to a question [24]. Tsampos and Marakakis, for example, developed a rule-based medical question answering system in Python using spaCy for natural language processing and Neo4j for graph database management [25]. They used Cypher queries to retrieve information from the graph database to answer user questions, and the system can handle complex questions by searching for relations between remote nodes and using synonyms to match nodes or paths [25]. Cairns et al. developed MiPACQ, a rule-based question answering system, by first retrieving candidate answer paragraphs using a paragraph-level baseline system based on the Lucene search engine [26]. The paragraphs were then re-ranked using a fixed formula that incorporated semantic annotations from the MiPACQ annotation pipeline [26]. This method utilized a scoring function that combined original paragraph scores with bag-of-words and UMLS entity components, ensuring that relevant paragraphs were prioritized for better question answering performance [26]. Information retrieval-based QA systems use keyword matching and ranking algorithms to retrieve documents or passages likely to contain the answer [27]. For example, Guo et al. developed a retrieval-based medical question answering system that efficiently retrieves answers using Elasticsearch and enhances them with semantic matching and knowledge graphs [28]. The system’s novel siamese-based answer selection architecture outperformed baseline models and systems in both Chinese and English datasets, demonstrating consistent improvements in quantification and qualification evaluations [28]. Deep learning-based QA systems have emerged as a more flexible and adaptable approach, leveraging techniques such as powerful neural network architectures to automatically learn to understand and respond to questions [29]. Yin et al. developed Evebot, a conversational system for detecting negative emotions and preventing depression through positive suggestions [30]. It uses deep-learning models including a Bi-LSTM for emotion detection and an anti-language sequence-to-sequence neural network for counseling [30].

While these traditional QA systems have been effective for certain types of questions and domains, they have several limitations. One major limitation is the reliance of rule-based approaches on predefined rules or keywords, which makes them less flexible and adaptable to new or complex questions [31]. These systems also struggle with understanding natural language queries and context, often leading to inaccurate or incomplete answers. Additionally, traditional QA systems are limited by the quality and coverage of their underlying knowledge base or document collection, which can affect the accuracy and relevance of their answers [32]. For deep learning-based QA systems, one major limitation is their dependency on large amounts of labeled training data [33], [34], [35], [36]. These systems require vast datasets to learn patterns in language and develop accurate models, which can be challenging and resource-intensive to obtain, especially for specialized domains or languages [33]. Additionally, deep learning-based QA systems may struggle with out-of-domain or adversarial examples, where the input falls outside the scope of the training data, leading to errors or inaccurate responses [29], [37], [38].

Another limitation of traditional QA systems is their inability to provide explanations or reasoning behind their answers [39]. These systems typically return a single answer without any supporting context or evidence, making it challenging for users to understand how the answer was derived [40], [41]. This lack of transparency can reduce user trust and confidence in the system, especially in critical applications such as healthcare or legal domains [42]. Overall, while traditional QA systems have been valuable in certain contexts, their limitations have led to the development of more advanced approaches

In recent years, the advent of powerful language models, such as the Generative Pre-trained Transformer (GPT), has revolutionized the field of natural language processing (NLP) and opened up new possibilities for conversational agents [35], [43], [44], [45]. GPT, developed by OpenAI, is a state-of-the-art deep learning model capable of generating human-like text based on the input it receives [35], [43], [44], [45]. The latest iteration, GPT-4, is distinguished by its ability to learn from vast amounts of text data, supported by its billions of parameters, enabling it to capture complex patterns in language and generate highly coherent and informative text [46], [47, p. 4], [48]. However, a significant challenge with GPT models, including ChatGPT, is their tendency to produce hallucinations or responses that, while plausible, are factually incorrect [49]. This issue has raised concerns about the reliability of these models, especially in critical applications such as healthcare [50]. To address this problem, researchers and developers are investigating the use of well-curated knowledge bases (KBs) to refine the models. By integrating authenticated and reliable information from KBs, the goal is to enhance the model’s capability to generate pertinent and accurate responses, thereby decreasing the risk of hallucinations. This has led to the development of chatbots and question answering systems powered by GPT that can provide information and assistance across various domains [48].

In the context of healthcare, the potential of GPT-powered question answering systems and chatbots is particularly promising [51]. Seenivasan et al. developed an end-to-end trainable Language-Vision GPT (LV-GPT) model to leverage GPT-based LLMs for Visual Question Answering (VQA) in robotic surgery [52]. The LV-GPT model extends GPT2 to process vision input (images) by incorporating a vision tokenizer and vision token embedding [52]. The model outperforms other state-of-the-art VQA models on public surgical-VQA datasets and a newly annotated dataset, demonstrating its effectiveness in capturing context from both language and vision modalities [52]. Shi et al. developed a GPT-based Question Answering System for Fundus Fluorescein Angiography (FFA) with an image-text alignment module and a GPT-based interactive QA module [53]. The system showed satisfactory performance in automatic evaluation and high accuracy and completeness in manual assessments, facilitating dynamic communication between ophthalmologists and patients for enhanced diagnostic processes [53]. Although GPT-powered question answering systems and chatbots in healthcare hold significant promise, we found that these systems exhibit hallucination issues because they use pre-trained GPT models directly without fine-tuning [53]. In the case of HPV vaccination, where inadequate information and misconceptions are prevalent, leveraging fine-tuning techniques with advanced GPT models can significantly enhance the accuracy and reliability of information provided. A GPT-powered chatbot, when properly fine-tuned, could play a crucial role in educating the public and increasing awareness about the importance of vaccination.

In this paper, we present the development and evaluation of a GPT-powered chatbot (VaxBot-HPV) designed to provide information and answer questions about the HPV vaccine. We also describe the design and implementation of the chatbot, its capabilities and limitations, as well as its potential impact on public health.

Overall, this paper highlights the potential of GPT-powered question answering systems and chatbots in healthcare, particularly in the context of HPV vaccination, and demonstrates how these systems can be leveraged to improve health literacy and promote vaccination uptake.

## 2. Materials and Methods

**Figure 1:**
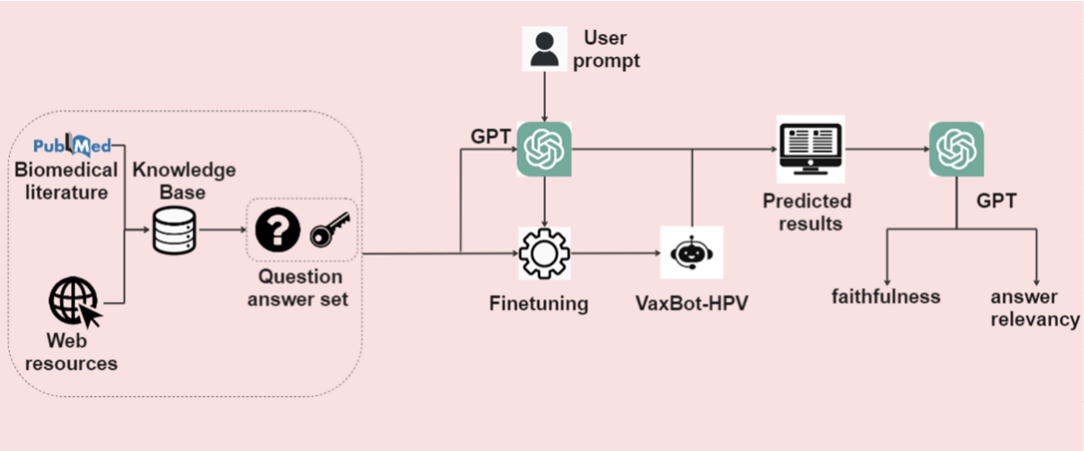
Overview of the framework.

The study is structured around three primary stages. Initially, we constructed a KB and collected question-answer pairs relevant to the HPV vaccine within the KB to develop the benchmark. Subsequently, we inferred answers for the questions in the test benchmark using both pretrained GPT models and GPT models fine-tuned on the benchmark. Finally, we assessed the results in terms of faithfulness and answer relevancy. Figure 1 shows the overview of the study framework.

### 2.1. KB and Gold Standard Construction

To build VaxBot-HPV, a chatbot designed to offer reliable information about the HPV vaccine, we first developed a KB deriving from peer-reviewed biomedical literature and web sources, resulting in a total of 451 documents on the HPV vaccine.

To construct the question-answer pairs, we extracted 202 pairs of frequently asked questions and their answers related to the HPV vaccine from the collected webpages in the KB. The gold standard of question-answer sets was meticulously reviewed by domain experts to ensure their relevance and accuracy.

### 2.2. Models

We utilized two state-of-the-art LLMs, GPT-3.5 and GPT-4, developed by OpenAI, as the key components of this study.

1. GPT-3.5: GPT-3.5 is the iteration in OpenAI’s series of large-scale language models, following the groundbreaking GPT-3. With an even larger model size (175 billion parameters) and enhanced capabilities, GPT-3.5 builds on the success of its predecessors in natural language processing (NLP) [54]. This advanced model exhibits impressive proficiency in understanding and generating human-like text, showcasing its potential for a wide range of applications including chatbots, content creation, and language translation [55].
2. GPT-4: GPT-4, the advancement in OpenAI’s renowned Generative Pre-trained Transformer series, marks a significant milestone in the field of natural language processing (NLP). With its remarkable increase to 170 trillion parameters, GPT-4 surpasses its predecessor, GPT-3, enabling it to tackle even more complex language tasks with improved accuracy and understanding [54]. This model represents a significant leap forward in NLP capabilities, holding the potential to revolutionize various fields, from conversational AI to content generation and beyond [55].

### 2.3. Experiment Setup

In this study, VaxBot-HPV, fine-tuned on GPT-3.5, was developed using question-answer pairs from both a knowledge base and GPT-generated questions. The question-answer pairs derived from the knowledge base were divided into 162 samples for training purposes and 40 for testing purposes. To enhance question diversity and ensure the generalizability of our findings, we employed GPT-4 models to generate 80 question-answer pairs. After careful review, we included 39 questions based on their relevance to the HPV vaccine and manually updated their answers for inclusion into our study. Among the GPT-generated questions, 28 question-pairs were randomly selected for training and the rest for testing.

The parameters of the VaxBot-HPV are outlined in Table 1. We used the following prompt to instruct the GPT models in answering the query:

“ You are an expert Q&A system that is trusted around the world.

Always answer the query using the provided context information, and not prior knowledge. Some rules to follow:

1. Never directly reference the given context in your answer.
2. Avoid statements like ‘Based on the context, …’ or ‘The context information …’ or anything along those lines.”

The prompts for GPT-4 to generate questions were as follows:

Using the provided context from referencing articles on HPV vaccine, formulate a question that captures an important fact from the context. Restrict the question to the context information provided. Please only output the question.

VaxBot-HPV’s development involved the performance comparison of various models, including GPT-3.5, as well as GPT-4, for each experimental set.

The experiments were carried out using a high-performance server containing 8 Nvidia A100 GPUs, each with a memory capacity of 80GB. This server configuration facilitated the effective training and evaluation of the models, ensuring the production of dependable and precise results.

### 2.4. Evaluation

The evaluation involved answer relevancy and faithfulness. Both are critical aspects in assessing the quality of generated responses. Answer relevancy gauges the extent to which the answers align with the questions, while faithfulness ensures factual accuracy, a fundamental requirement for reliable information retrieval. These metrics collectively provide a comprehensive evaluation of the model’s performance in understanding and responding to user queries. Additionally, evaluations were conducted to thoroughly assess the system’s effectiveness.

The assessment of all outcomes was carried out using the Ragas metrics, which are GPT-supported measures widely adopted in NLP tasks to evaluate the quality of generated text [56]. Specifically, the RAGAS metrics calculate answer relevancy and faithfulness through a detailed process. For answer relevancy, the ground truth answer and the generated answer are vectorized using the specified embedding model, and their cosine similarity is computed to determine alignment [56]. For answer faithfulness, the process involves quantifying factual correctness by identifying true positives (facts present in both the ground truth and the generated answer), false positives (facts present in the generated answer but not in the ground truth), and false negatives (facts present in the ground truth but not in the generated answer) [56]. The F1 score is then used to quantify correctness based on these values [56]. A weighted average of factual correctness and semantic similarity provides the final score [56].

## 3. Results

Table 2 illustrates the automatic performance evaluation of different GPT models in answer relevancy and faithfulness on the questions extracted from the KB. The results indicate that the VaxBot-HPV outperformed both the GPT-3.5 andGPT-4 models in terms of answer relevancy, achieving a score of 0.85 compared to 0.80 and 0.83, respectively. Similarly, the VaxBot-HPV exhibited higher faithfulness, scoring 0.97, compared to 0.92 for the GPT-3.5 model and 0.91 for the GPT-4 model. These results suggest that fine-tuning the GPT-3.5 model leads to improved performance in both answer relevancy and faithfulness compared to using the models in their pretrained states.

Table 3 presents the performance evaluation of different GPT models in terms of answer relevancy and faithfulness on questions generated by GPT-4. The GPT-3.5 model achieved an answer relevancy score of 0.80 and a faithfulness score of 0.90. In comparison, VaxBot-HPV showed improved performance with an answer relevancy score of 0.85 and a faithfulness score of 0.96. These results highlight the benefits of fine-tuning the GPT model, demonstrating its broader generalizability, applicability and robustness.

**Figure 2:**
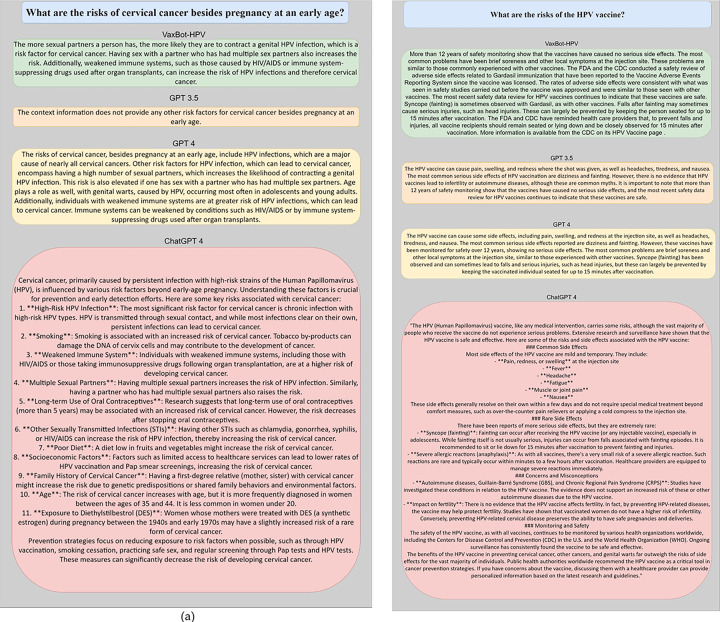
Samples of questions and answers (a) GPT generated question (b) question in the test benchmark over systems

Figure 2 shows two samples of questions and its generated questions by the four systems. We selected two questions. One question (“What are the risks of cervical cancer besides pregnancy at an early age?”) is generated by GPT, and another question (“What are the risks of the HPV vaccine?”) is from the test benchmark. VaxBot-HPV demonstrates an advantage in providing comprehensive and accurate responses to health-related inquiries compared to other systems. For instance, when asked about the risks of cervical cancer besides early pregnancy, VaxBot-HPV effectively listed multiple risk factors, including having multiple sexual partners, weakened immune systems, and specific health conditions. In contrast, the GPT-3.5 failed to identify any additional risk factors, while the GPT-4 provided information not directly to the question, such as “genital warts occurred most in adolescents and young adults”, which could be misleading. Additionally, ChatGPT, although comprehensive, was not succinct and failed to answer the question directly. Furthermore, when it comes to the question ”What are the risks of the HPV vaccine?”, VaxBot-HPV effectively summarized over 12 years of safety monitoring, highlighted common and rare side effects, and provided actionable advice on preventing fainting-related injuries, all while maintaining a clear and concise format. In contrast, the GPT-3.5 and GPT-4, though accurate, lacked depth, information sources and reassurance, merely listing side effects without addressing common myths or providing detailed context. ChatGPT-4, despite its comprehensiveness, often failed to deliver succinct answers, resulting in verbose responses that lacked focus. These examples illustrate that VaxBot-HPV not only enhances the specificity and clarity of responses but also ensures that users receive accurate, reliable and actionable health information efficiently.

## 4. Discussion

The development and evaluation of VaxBot-HPV, a chatbot designed to provide information and answer questions about the HPV vaccine, demonstrates the potential of advanced language models, particularly GPT-3.5 and GPT-4, in healthcare applications. Compared to traditional QA systems, VaxBot-HPV leverages the capabilities of GPT models, especially after fine-tuning, to generate relevant and accurate responses to user queries.

VaxBot-HPV has a substantial advantage over existing pre-trained GPT models. This extensive pre-training allows VaxBot-HPV to have a deeper understanding of language and context, enabling it to provide more relevant and accurate answers to user queries. Unlike rule-based systems, which rely on predefined rules and patterns, and retrieval-based systems, which use keyword matching and ranking algorithms, VaxBot-HPV’s pretrained model allows it to generate responses based on a broader understanding of the topic. This capability enhances the chatboťs ability to address a wide variety of questions and provide more informative and helpful responses to users. Moreover, VaxBot-HPV allows the answers to be dynamically generated, potentially offering more tailored responses to users compared to standard, one-size-fits-all answers. The fine-tuning process further enhances VaxBot-HPV’s performance, particularly in the context of HPV vaccination, by adapting it to the specific domain. This adaptation improves answer relevancy and faithfulness, addressing common issues of ChatGPT such as hallucinations, where the model generates plausible but inaccurate responses. By fine-tuning on a dataset specific to HPV vaccination, VaxBot-HPV can learn the nuances of the topic, including relevant terminology, common misconceptions, and specific concerns that users may have. This specificity allows the chatbot to provide more accurate and tailored responses, increasing its overall effectiveness in addressing user queries related to the HPV vaccine. Furthermore, the fine-tuning process helps mitigate bias and misinformation that may be present in generic language models, ensuring that VaxBot-HPV provides reliable and trustworthy information to users seeking information about HPV vaccination. Additionally, the specific fine-tuning, which includes context in addition to question-answer pairs, enables VaxBot-HPV to extend beyond just answering questions. These models can also provide explanations or reasoning behind their answers, increasing transparency and user trust. This feature is particularly important in healthcare applications, where understanding the rationale behind medical advice is crucial for informed decision-making.

In terms of evaluations, incorporating multiple sources, including questions generated by GPT models, strengthens the credibility and reliability of our findings regarding VaxBot-HPV’s performance. By leveraging questions from diverse sources, we were able to assess the chatboťs ability to handle a wide range of queries beyond those explicitly included in the knowledge base. This comprehensive evaluation approach not only ensures the robustness of our results but also demonstrates VaxBot-HPV’s versatility in addressing various user inquiries. Overall, the use of multiple evaluation metrics underscores the effectiveness and adaptability of VaxBot-HPV in providing reliable information and support to users.

While VaxBot-HPV demonstrates promising performance, there are several limitations to consider. First, the chatboťs effectiveness is contingent on the quality and comprehensiveness of the underlying knowledge base. Incomplete or inaccurate information in the KB could lead to erroneous or insufficient responses from the chatbot. Additionally, the chatboťs reliance on text-based interactions may limit its accessibility to individuals with visual or cognitive impairments who may benefit from alternative communication methods. Moreover, the inclusion of manual evaluations is needed to provide a holistic assessment of the chatboťs performance, enhancing the depth and accuracy of our conclusions. Furthermore, the evaluation of VaxBot-HPV was primarily based on its performance in answering questions, overlooking other aspects of user interaction such as ease of use, user satisfaction or engagement. Finally, the generalizability of our findings may be limited to the specific domain of HPV vaccination and may not extend to other healthcare contexts.

Future research could focus on several areas to enhance the capabilities and impact of VaxBot-HPV. First, expanding the knowledge base to include a broader range of topics related to HPV vaccination and addressing emerging concerns or misconceptions could improve the chatboťs effectiveness and relevance. Second, integrating multimedia capabilities, such as image or video recognition, could enhance the chatboťs ability to provide information and support in a more interactive and engaging manner. Third, incorporating feedback mechanisms to gather user input and improve the chatboťs responses over time could enhance its usability and user satisfaction. Furthermore, exploring the integration of VaxBot-HPV with existing healthcare systems or platforms could facilitate its adoption and integration into clinical workflows, potentially improving access to information and promoting HPV vaccination uptake. Fourth, extending the chatbot to cover other types of vaccines and medical domains could broaden its applicability and utility, making it a more versatile tool for addressing public health concerns. Lastly, we need to add a user interface to VaxBot-HPV to make it more accessible and user-friendly, enhancing the overall user experience and encouraging more people to use the chatbot for reliable information on HPV vaccination and related topics.

## 5. Conclusions

In conclusion, the development of VaxBot-HPV demonstrates the potential of GPT-powered chatbots in healthcare, particularly in promoting vaccination uptake and addressing common concerns and misconceptions. The study also underscores the importance of leveraging advanced language models and fine-tuning techniques in healthcare chatbot development. The efficacy of VaxBot-HPV highlights the transformative impact of such technologies on medical education, healthcare communication and information dissemination.

## Figures and Tables

**Table 1:** Parameters of VaxBot-HPV.

Parameter	Value
n_epochs	2
batch_size	1
learning_rate_multiplier	1
temperature	0.3
context_window	2,048
Token limit	4,096

**Table 2:** Performance evaluation of different GPT models in answer relevancy and faithfulness on the questions extracted from the knowledge base.

Model	Answer Relevancy	Faithfulness
GPT-3.5	0.80	0.92
VaxBot-HPV	0.85	0.97
GPT-4	0.83	0.91

**Table 3:** Performance evaluation of different GPT models in answer relevancy and faithfulness on the questions generated by GPT-4.

Model	Answer Relevancy	Faithfulness
GPT-3.5	0.80	0.90
VaxBot-HPV	0.85	0.96

## Data Availability

Dataset available on request from the authors.
